# Data mining of accidents in Spanish underground mines in the period 2003–2021 caused by a collision with a moving object

**DOI:** 10.1016/j.heliyon.2024.e24716

**Published:** 2024-01-19

**Authors:** Lluís Sanmiquel, Josep M. Rossell, Marc Bascompta, Carla Vintró, Mohammad Yousefian

**Affiliations:** a Department of Mining Engineering, Industrial and ICT, Polytechnic University of Catalonia (UPC), Avenue Bases de Manresa, 61-73, 08242, Manresa (Barcelona), Spain; b Department of Mathematics, Polytechnic University of Catalonia (UPC), Avenue Bases de Manresa, 61-73, 08242, Manresa (Barcelona), Spain; c Department of Management, Polytechnic University of Catalonia (UPC), Avenue Bases de Manresa, 61-73, 08242, Manresa (Barcelona), Spain

**Keywords:** Data-mining, Mine drift, Type of accident (TA), Underground mining, Weka

## Abstract

Underground mining is currently one of the Spanish economic sectors with the worst accident rates. Besides, the most frequent type of accident, and with the most serious consequences, is the one in which the injured worker is hit by a moving object. For this reason, this study focuses on the analysis of this type of accident, divided into 3 subgroups to better understand the behavioural patterns. Data mining techniques were applied using the Apriori algorithm to extract as much information as possible about the genesis of these accidents. Similarly, each subset of accidents was processed in two different ways to improve the data analysis, depending on the causal variables used in each case, so that a study of six different scenarios was carried out. The five best association rules or behaviour patterns for each of the six scenarios are shown as a function of their frequency for each rule with 1–4 causal variables.

## 1 Introduction

In the last 40 years, the occupational accident rate in the Spanish mining sector has substantially improved, based on the evolution of the Loss of Accident Frequency Index (LTIFR) as the Ministry of Labor has claimed that the LTIFR has evolved from a 7 times higher ratio than the total economic sectors in 1980 to 2.9 times in 2018. Despite this improvement, the mining sector continues to be considerably dangerous in Spain, which is also common in most countries [1]. In fact, many studies have analysed the reasons of higher occupational risk in the mining sector over time [[2], [3], [4], [5], [6], [7], [8]], implying that some features such as environmental conditions with a significant presence of dust, humidity, falling rocks, or the usage of heavy equipment have an important influence on the number and severity of accidents. Furthermore, specific workplace environmental conditions are considerably worse in underground mining than in open pit mining, as can be seen in Table 1.

**Table 1:** Lost time injury frequency rate in mining and total economic sectors in Spain.

Year	Underground Mining	Open pit Mining	Total Economic Sectors
2003	232.33	94.05	37.60
2004	200.79	86.92	36.30
2005	172.74	72.96	35.90
2006	160.49	68.91	36.62
2007	176.75	70.46	36.00
2008	237.29	64.46	31.89
2009	240.80	50.14	26.47
2010	209.75	43.99	25.11
2011	197.83	40.93	22.81
2012	185.04	35.54	18.64
2013	183.12	31.36	19.80
2014	221.47	33.89	20.50
2015	217.84	36.43	21.50
2016	195.77	34.89	22.30
2017	119.81	32.55	22.50
2018	135.66	39.61	22.50
2019	161.37	37.64	22.30
2020	140.31	27.71	19.50
2021	89.66	33.16	21.20

The LTIFR of underground and open pit mining in Spain has been calculated using official data, from the Spanish Ministry of Labor and Social Economy. However, not all occupational accidents in the mining sector can be attributed solely to the challenging environmental conditions. Inadequate risk prevention management systems have been considered an important factor in accidents in several studies over time [[9], [10], [11], [12], [13], [14]]. For instance, Wang and Meng [15] stated that disregard of regulations and procedures was the main cause of nearly 80 % of disasters in Chinese coal mines. Besides, Düzgün and Leveson [16] revealed that one of the weaknesses of the health and safety system in Turkish mines was the lack of a proper legislative framework. Ivaz et al. [17] also highlighted that the analysis of injuries with fatal outcomes in underground coal mines from Serbia showed that changes in health and safety legislation, based on the attitude towards safety, led to a significant decrease in fatal injuries in the sector. The recognition of hazards encountered in the whole mining process should be the basis for safety management decisions. The primary objective of these decisions should be to identify and assess problematic areas and weaknesses in the health and safety system of mining companies [18].

When the Spanish mining sector is compared to other countries, work accident indexes remain considerably higher than those of some countries. For instance, the LTIFR was 19 times higher than the State of Queensland (Australia) in 2019 and 5.7 times higher than the USA in 2021, according to the Queensland Mines and Quarries Safety Performance, and Health Report and the National Institute for Occupational Health and Safety in United States, respectively. Thus, it is crucial to determine the major occupational hazards in the Spanish mining sector, as well as their factors and causes, to implement better policies and, therefore, improve the safety conditions. In this sense, accident models are important for risk assessment in terms of preventing dangerous deviations that may lead to similar accidents in the future, as well as helping in the identification of the risks and how the accidents happen [19,20]. It is well-known that statistical methods are the most common tools for the analysis of injuries [21], extract rates [22], correlate different variables [[23], [24], [25]], predict accidents [26], or compare economic sectors [27], among many other possibilities. However, the need to process data, such as occupational accident databases, has grown over the years, requiring the use of appropriate tools to handle large amounts of complex interrelated information. In this context, data mining techniques are an appropriate and validated approach to studying the genesis of large databases of occupational accidents [28,29], allowing the transformation of the accident causes into valuable information and, consequently, identifying the main accident patterns and the sequence between them. As a result, it will also be possible to avoid potential losses that reduce the performance of production systems such as a decrease in production efficiency, loss of trained workforce, loss of work days, etc. [30].

In addition, data mining techniques can be developed using different algorithms, such as the Apriori algorithm, which is considered one of the most common and effective for extracting frequent item sets for association rules [31]. Asur et al. [32] use the Apriori algorithm to determine the visual preferences of the observers for different landscape types, while De Carvalho et al. [33] identify the processes and actors involved in the regulatory processes of the energy sector, mapping the relationships between these processes and actors through the Apriori algorithm, using a survey as initial information. Akbas et al. [34] obtained association rules by applying the Apriori algorithm on 18 genetic health indicators from 225 patients, facilitating the physician's diagnosis with these association rules. Applying the same process, Mohajeri et al. [35] uses information structured into 14 variables from 17,846 accidents in the construction sector in Iran, whereas Mohamad et al. [36] find the rules of association between the 34 classes of 6 predictor variables, selected from an analysis of previous research, using 167,820 road accidents in Thailand.

Bayesian networks encode probabilistic relationships between variables of interest in data mining studies, which brings advantages to data modeling when used in conjunction with statistical techniques [37]. A Bayesian network can be used to learn causal relationships and, therefore, to understand the problem domain and predict the consequences of an intervention [38]. It is important to note that Bayesian networks can generally simulate the expert's quality assessment procedure to a satisfactory degree and accelerate the quality estimation process of the studied topic [39]. Likewise, the use of data mining techniques and/or Bayesian network are more sensitive in detecting associations among categorical variables than other methods [37,38]. Therefore, this methodology can prove highly valuable in obtaining dependable conclusions for the decision-making process concerning workplace safety issues as it has been applied in many other scientific fields, such as civil engineering [40], road traffic safety [36,41,42], the interrelation between hygienic workplace conditions and occupational accidents [43] or construction and mining [28,29,[44], [45], [46]]. Following this context, some studies have inferred a similar pattern in terms of LTIFR in underground and open pit mining, with even greater differences over time, using data mining techniques to identify different patterns of injury behaviour or standards in surface and underground mining [46].

The current study uses data mining techniques through the Apriori algorithm to find rules of association between the predictor variables. These variables were selected from the official Spanish accident database, which provides the most detailed and accurate information about the accident's circumstances. Previous studies used a similar approach [28,46], but predictor variables were selected using Bayesian networks. The study is focused on the analysis of the most relevant type of accident in underground mining according to the official database from Spain, accidents caused by impact or collision with a moving object. Findings from this research can provide mining companies with vital information to make the necessary and appropriate technical and safety investments to reduce the number of accidents in their underground mining operations. This will not only achieve the main aim of improving the safety levels of workers in Spanish underground mines but will also have a positive economic impact by reducing the direct and indirect costs of accidents to companies. In addition, it will contribute to the alignment of the mining sector with the Sustainable Development Goals (SDGs), in particular SDG 3, Good health and well-being, and SDG 9, Industry, innovation and infrastructure. This will also mean an improvement in the corporate social responsibility of the mining sector, which can have a positive socio-economic impact [47].

## 2 Material and methodology

### 2.1 Study population

Data were obtained from the annual digital database of accidents from the Spanish Ministry of Labour and Social Economy, for the period between 2003 and 2021. An initial classification was done to rank them from most to least frequent cause of the accident, the 3 most frequent types of accidents are type 4, 7 and 3, with 15,528, 12,588 and 5991 accidents, respectively. Accident type 4 includes accidents caused by impact or collision with a moving object. Accident type 7 is defined as accidents caused by physical overexertion; and accident type 3 consists of accidents caused by impact or collision with stationary objects, where the worker moves vertically or horizontally. The different types of accident are detailed in section 2.

It is also noteworthy that type 4 is not only the most frequent type of accident, 15,528 accidents with at least one day of sick leave, but also the one that has caused the highest number of fatalities. Thus, the study is focused on the analysis of this type of accident, with the idea of finding behavioural patterns, which are defined as a combination of classes of causal variables, resulting in a response variable that represents a type 4 accident in all cases. Itinerary accidents were not included in this research. Furthermore, this study is based on the classification of the total number of accidents of type 4 into the three following subgroups, each one consisting of the type of accident (TA); an abnormal event immediately preceding the accident, called deviation or previous cause (PC); and a material agent (MA).

a) Subgroup 1:
• TA from group 4 (TA4), hit or collision with a moving object, based on collisions or impacts due to the fall or detachment of an object from an upper part.
• PC characterised by a slip, fall or collapse of a material object falling on the worker from an upper position or a lateral position.
  • MA related to the underground tunnels or drifts.

Subgrup 1 includes those accidents that are more related to geological or geotechnical causes, since the material agent is always an element detached from a tunnel or drift of a mine. This combination of circumstances resulted in 781 accidents, of which 765 were minor, 8 serious, and 8 fatal. In addition, these 781 accidents produced a total of 73,263 lost working days, giving a rate of 93.81 lost working days per accident.

b) Subgroup 2:
• TA from group 4, hit or collision with a moving object, based on jolts or impacts due to the fall or detachment of an object from an upper part.
• PC characterized by a slip, fall or collapse of a material object falling on the worker from an upper position or a lateral position.
• MA not related to the underground tunnels or drifts.

This combination of circumstances resulted in 4906 accidents, of which 4855 were minor, 46 serious and 5 fatal. In addition, these 4906 accidents produced a total of 163,606 lost work days, giving a rate of 33.35 lost working days per accident.

c) Subgroup 3. All other type 4 accidents not covered by the two previous subgroups. Having a total amount of 9841 accidents, of which 9774 were minor, 63 were major and 4 were fatal. The 9841 accidents resulted in a total of 287,850 lost work days, giving a rate of 29.25 lost work days per accident.

Therefore, in the previous subgroup described the same analysis was carried out using data mining techniques with Weka software for the accident databases. In the three subgroups, the response variable was the same and unique (accidents type of class 4), and in subgroups 1 and 2 the deviation or the previous cause was also the same and unique (an abnormal event immediately preceding the accident characterised by a slip, fall or collapse of a material object falling on the worker from an upper or lateral position). In addition, subgroup 1 also includes the material agent of the same class (a physical agent associated with underground tunnels or drifts). It is important to note that a causal variable can be ignored in a Weka study if that variable has a single class or category. Furthermore, the accidents in subgroup 3 are the only ones where there is no class or category condition for the PC, as in subgroups 1 and 2, and for the material agent as in subgroup 1.

### 2.2 Statistical procedure

Storing and using a database is a quite common procedure, while analysing the meaningful dataset for a specific purpose can be difficult. In this regard, the concept of data mining is used to find effective ways of processing data with the ability to detect behavioural patterns from large data sets.

Although there are many software packages available on the market to work with mining data, Waikato Environmental for Knowledge Analysis (WEKA) software, v.3.9.6, has been used. It is an open-source software widely used in this type of research [28,32,33,46,48], with a set of tools very useful for performing data pre-processing, classification, clustering, association rules, and visualisation operations [[49], [50], [51]]. Likewise, Weka is very popular for implementing machine learning algorithms.

**Fig. 1:**
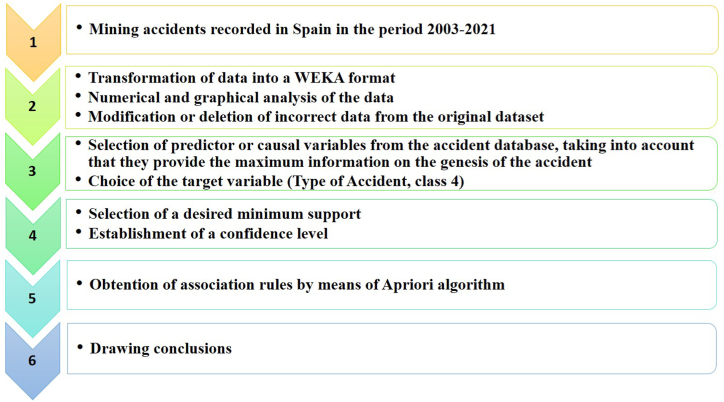
Statistical procedure decomposed in six steps.

In this study, the data mining process takes place in six steps: (1) database obtaining, Spanish mining accidents for the period 2003–2021; (2) data cleaning and transformation, deleting incorrect data, converting data formats, and ensuring data consistency. In addition, extraction of a numerical and graphical descriptive study of data using standard methodologies; (3) prior selection of predictor variables from the initial database, as well as the response variable Type of Accident (TA4). In this case, and considering our previous experience with this topic [1,14,28,46], those variables that could be mostly involved with the response variable have been chosen; (4) establishment of a confidence level and minimum support; (5) generation of association rules after parameters configuration; and (6) evaluation of results through association rules and extraction of conclusions to make future predictions (Fig. 1).

Regarding point (4) described above, confidence level is defined as the proportion of instances that are encompassed by the premise of a rule which are also covered by the consequence. It measures the reliability of the inference stemming from a given rule. Considering a specific association rule X → Y, the higher the confidence, the more likely it is that Y will be present in transactions containing X. Confidence also provides an estimate of the conditional probability of Y given X. However, in this study, since the response variable TA4 only has a single class, all the association rules obtained will have a confidence level equal to 1.0, 100 %, since the target variable is the same accident type in the three accident subgroups considered.

Support is the percentage of the complete dataset covered by a rule. It is obvious that with high support the association rules that contain the most frequent predictor variables appear, but less frequent rules that also involve serious accidents cannot be detected. Regarding the minimum supports, different values have been considered depending on the established scenarios and minimum supports of 35 %, 10 %, and 8 % have been established.

Concerning point (5), an association rule helps identify undiscovered relationships that can be used for forecasting and decision-making. As previously stated, the Apriori algorithm is the most used for mining association rules, being an efficient algorithm for finding all frequent item sets [26,31]. First, the database is scanned to identify all item sets with support values equal to or greater than a predetermined minimum value. Apriori then uses an iterative technique known as level-wise search, in which k sets of items are used to explore (k +1) sets of items. Apriori association rule learner has been used because it combines confidence and coverage into a single measure of predictive accuracy.

After a prior selection of all the variables available in the database, 13 possible causal or predictor variables have been selected, according to the importance of what they can contribute to the knowledge of the genesis of accidents for the materialisation of a given type of accident. In order to facilitate further studies, variables have been grouped into classes or categories.

1. Age (A): Age of the injured worker at the moment of the accident. A1 = [16,24], A2 = [25,29], A3 = [30,34], A4 = [35,39], A5 = [40,44], A6 = [45,54], A7 = [55, →].
2. Contract (C): Type of employment contract. C1 = permanent and full-time; C2 = permanent and part-time; C3 = temporary and full-time; C4 = temporary and part-time.
3. Contractual Status (CS): CS1 = the injured worker belongs to the company that owns the mine (own worker); CS0= The injured worker does not belong to the company that owns the mine (external worker).
4. Day Hour (DH): Time period in which the accident occurred. DH1=(0,6], DH2= (6,10], DH3=(10,14], DH4=(14,18], DH5=(18,24].
5. Experience (E): Months of experience in this type of work. E1 = [0,12], E2 = [1,30], E3 = [31,60], E4 = [61,120], E5 = [121,180], E6 = [181,240], E7 = [241,→].
6. Material Agent (MA): MA1 = surfaces or circulation areas at the same level floor (inside or outside); MA2 = buildings, constructions, surfaces in height or depth (interior or exterior); MA3 = underground tunnels or drifts; MA4 = material distribution devices, power supply devices, pipelines; MA5 = engines, energy transmission and storage devices; MA6 = hand tools; MA7 = machines and equipment, mobile or stationary; MA8 = moving, transport and storage devices; MA9 = land vehicles; MA10 = other transport vehicles; MA11 = materials, objects, products, machine elements, fractures, dust; MA12 = other material agents.
7. Physical Activity (PA): Type of physical activity that the worker was making at the time of the accident. PA1 = machine operations; PA2 = working with hand tools; PA3 = driving or being in a conveyance; PA4 = manipulation of objects; PA5 = manual handling of loads; PA6 = performing a movement; PA7 = others.
8. Preventive Organization (PO): Preventive organization system of the company to which the injured worker belongs. PO1 = the employer; PO2 = designated workers; PO3 = own prevention service; PO4 = joint prevention service; PO5 = external prevention service; PO6 = without prevention service.
9. Previous Cause or Deviation (PC): Existence of an anomalous event just before the accident. PC1 = electric problem, explosion, fire, PC2 = overflow, overturn, leak, spill, vaporization or emanation; PC3 = break, rupture, burst, slip, fall, collapse of a material agent; PC4 = loss of control (total or partial) of the working machinery; PC5 = falls/tumbles of a person; PC6 = body movement without physical effort; PC7 = body movement with physical effort; PC8 = other causes.

It is possible to distinguish in PC3 between slipping, falling and collapsing of a material agent on the one hand, and breaking, rupturing and bursting of a material agent on the other.

10. Risk Evaluation (RE): It indicates whether the company carried out a workplace risk assessment where the accident occurred: RE0 = no; RE1 = yes.
11. Size (S): Number of workers in the mine work centre where the accident occurred. S1 = [0,9], S2 = [10,19], S3 = [20,49], S4 = [50,99], S5 = [100,499], S6 = [500,→].
12. Week Day (WD): Day of the week that the injury happened. WD1 = Monday; WD2 = Tuesday; WD3 = Wednesday; WD4 = Thursday; WD5 = Friday; WD6 = Saturday; WD7 = Sunday.
13. Worked Hours (WH): It indicates how many hours the employee worked before the accident. WH1=(0,1], WH2=(1,4], WH3=(4,8], WH4=(8,10], WH5=(10,12], WH6=(12,→].

The response variables are classified as Type of Accident (TA). The variable explains the cause of the accident and it has been divided into nine categories: TA1 = electric contact, fire, contact with hazardous substances, drowning; TA2 = being buried under a solid object; TA3 = impact or collision with stationary object; TA4 = hit or collision with a moving object; TA5 = shock or blow caused by a falling or dislodged object; TA6 = contact with a sharp or pointed object; TA7 = being trapped, crushed or amputated; TA8 = physical overexertion, psychological trauma, exposure to radiation, noise, light or pressure; TA9 = other types of accidents. However, as indicated above, TA4 is the only class considered in this study.

The next step is the selection of the target or response variable, using the other 13 possible causal variables as predictors, for each subgroup of accidents considered in the Spanish underground mining industry, during the period 2003–2021. As previously mentioned, the only response variable used in this study is the type 4 accident.

The 13 causal variables used in this study are those which, based on the experience of the authors, are considered to have the greatest influence on the genesis of a given type of accident. Thus, only those variables that provide some kind of information on the previous circumstances of the accident were considered. Consequently, variables that provide some kind of information about the consequences of the accident, such as the type of injury, the part of the body injured, and the number of days lost from work, have not been considered. Likewise, the 13 causal variables have been divided into two categories in order to carry out the analysis with Weka for each of the accident subgroups indicated in section 2.1.

On the one hand, there is a first set of five causal variables that are characterised by a more documentary and administrative nature, strongly influenced by the general characteristics of the Spanish underground mining industry, as well as by the Spanish legislation that directly or indirectly regulates all the aspects related to the prevention of occupational risks in mining. As a result, these variables tend to show little variability between different mines, workplaces, different groups of accidents, etc. If this study had included accident groups not only pertaining to underground mining, but also to quarries or rock and mineral processing plants, the differences would probably have been larger. The five causal variables are Contract (C), Contract Status (CS), Risk Evaluation (RE), Prevention Organisation (PO) and Size (S).

On the other hand, the causal variables are grouped in a second group of eight variables that define aspects related to the activity carried out by the worker at the time of the accident, such as the material agent with which he/she was injured, the characteristics of the worker, the time and day of the accident and the anomalous event that occurred just before the accident and which was the determining factor in the accident. These variables may show greater variability between mines, workplaces, workers, and different times of the day and week. The eight causal variables in this block are: Age (A), Day Hour (DH), Experience (E), Physical Activity (PA), Weekday (WD), Worked Hours (WH), Material Agent (MA), and Deviation or Previous Cause (PA).

Subsequently, two approaches were considered for each of the three accident subgroups, one with the variables of the first block, and the other with the variables of the second block, reaching a total of six scenarios. This is done in order to be able to extract more behaviour patterns in the genesis of accidents in a given scenario and, thus, to have a better understanding of the circumstances of the accidents.

## 3 Results and discussion

Based on the classification defined in section 2, scenarios 1.1 and 1.2 are based on accidents from subgroup 1, scenarios 2.1 and 2.2 on accidents from subgroup 2, and scenarios 3.1 and 3.2 on accidents from subgroup 3. The difference between the scenarios for the same subgroup of accidents is in the causal variables considered, as described at the end of section 2.2.

The association rules are classified according to the number of causal variables. Thus, rules with one, two, three, and four variables are considered whenever possible. In addition, the causal variables Previous Cause (PC) and Material Agent (MA) will not appear in the association rules of scenarios 1.1 and 1.2 because, as indicated in section 2.1, the grouping of accidents in subgroup 1 only considers accidents with a previous cause of class 3 (PC3) and an agent material of class 3 (MA3). However, they are also included in the association rules, because they define important characteristics of the accident genesis. The same applies to scenarios 2.1 and 2.2, but only for PC3. Similarly, in scenarios 1.1, 1.2, 2.1, and 2.2, the previous cause or abnormal event of class 3 (PC3) only covers situations related to the slip, fall, or collapse of a material agent, whereas scenarios 3.1 and 3.2 mainly cover situations related to the break, rupture or burst of a material agent.

It has been necessary to set a minimum support in the Weka process in order to find the 5 best association rules with three or four causal variables. Thus, this support has been able to be set to a minimum of 35 % for scenarios 1.1, 2.1, and 3.1, while for scenarios 1.2, 2.2, and 3.2 it has been only 10 % for the first two scenarios and 8 % for the third. This difference between scenarios 1.1, 2.1, and 3.1 with respect to scenarios 1.2, 2.2, and 3.2 is a consequence of the differences between the characteristics of the two groupings of causal variables established in this study, as discussed in section 2.2.

### 3.1 Scenarios 1.1, 2.1 and 3.1

**Fig. 2:**
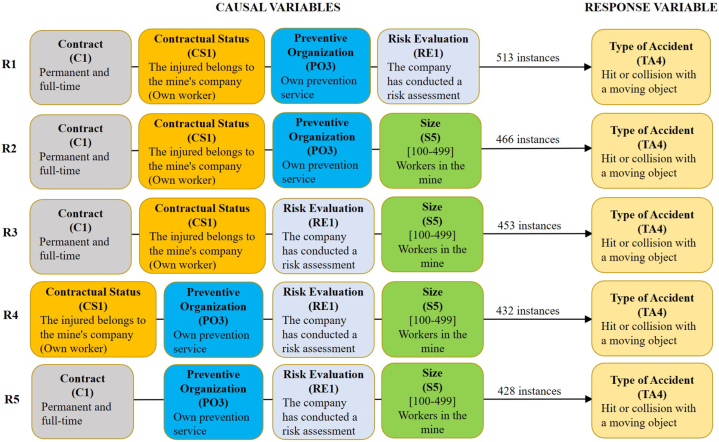
The 5 best association rules (R1 to R5) with four causal variables for scenario 1.1. Previous cause (PC) = 3: Slip, fall, or collapse of a material object falling on the worker from an upper or a lateral position. Material agent (MA) = 3: Underground tunnels or drifts. Minimum support is required to find all sets of frequent items = 35 %.

**Fig. 3:**
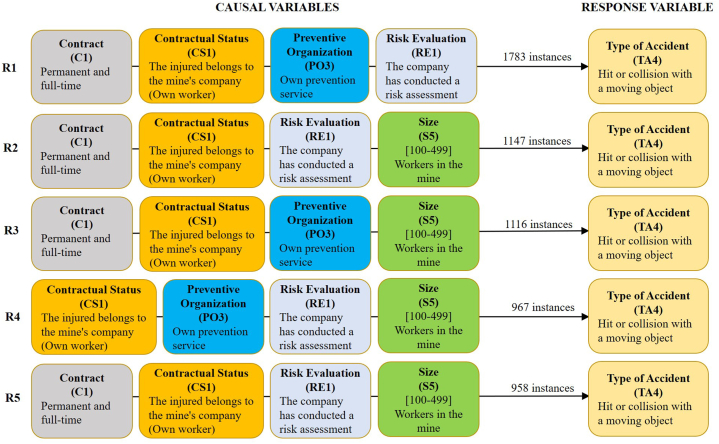
The 5 best association rules (R1 to R5) with four causal variables for scenario 2.1. Previous Cause (PC) = 3: Slip, fall, or collapse of a material object falling on the worker from an upper or a lateral position. Material Agent (MA)≠3: Underground tunnels or drifts. Minimum support is required to find all sets of frequent items = 35 %.

**Fig. 4:**
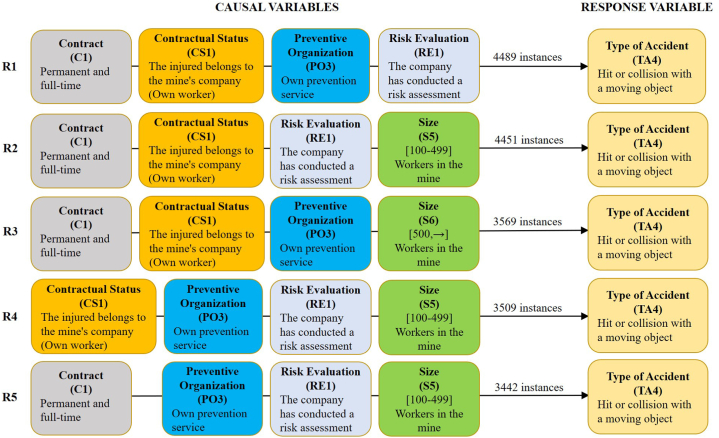
The 5 best association rules (R1 to R5) with four causal variables for scenario 3.1. This scenario includes the accidents of type 4 not covered by subgroups 1 and 2, indicated in section 2.1. Minimum support is required to find all sets of frequent items = 35 %.

These scenarios include the specific accidents to each of their subgroups with the causal or predictive variables Contract (C), Contract Status (CS), Risk Evaluation (RE), Prevention Organisation (PO), and Size (S). Table 2, Table 3, Table 4 summarise the 5 best association rules for one, two, three, or four causal variables and Fig. 2, Fig. 3, Fig. 4 show the 5 best rules with the combination of the four causal variables. The numbers in brackets indicate the number of accidents or instances in which the combination of classes of causal variables in question occurs.

**Table 2:** The best five first association rules for one, two, three, or four causal variables for scenario 1.1 with 781 accidents or instances.

	R1	R2	R3	R4	R5
One causal variable	CS1 (736)	C1 (722)	RE1 (667)	PO3 (590)	S5 (565)
Two causals Variables	C1+CS1 (693)	CS1+RE1 (633)	C1+RE1 (619)	CS1+PO3 (586)	C1+PO3 (571)
Three causals variables	C1+CS1+RE1 (596)	C1+CS1+PO3 (570)	CS1+PO3+RE1 (526)	C1+CS1+S5 (521)	C1+PO3+RE1 (514)
Four causals variables	C1+CS1+PO3+RE1 (513)	C1+CS1+PO3+S5 (466)	C1+CS1+RE1+S5 (453)	CS1+PO3+RE1+S5 (432)	C1+PO3+RE1+S5 (428)

**Table 3:** The best five first association rules for one, two, three, or four causal variables for scenario 2.1 with 4906 accidents or instances.

	R1	R2	R3	R4	R5
One causal variable	CS1 (4163)	C1 (3640)	RE1 (3236)	PO3 (2476)	S5 (1868)
Two causals Variables	C1+CS1 (3208)	CS1+RE1 (2775)	C1+RE1 (2449)	CS1+PO3 (2430)	C1+PO3 (2332)
Three causals variables	C1+CS1+RE1 (2241)	CS1+PO3+RE1 (1840)	C1+PO3+RE1 (1787)	C1+CS1+S5 (1492)	C1+RE1+S5 (1214)
Four causals variables	C1+CS1+PO3+RE1 (1783)	C1+CS1+RE1+S5 (1147)	C1+CS1+PO3+S5 (1116)	CS1+PO3+RE1+S5 (967)	C1+CS1+RE1+S5 (958)

**Table 4:** The best five first association rules for one, two, three, or four causal variables for scenario 3.1 with 9841 accidents or instances.

	R1	R2	R3	R4	R5
One causal Variable	CS1 (8766)	RE1 (8113)	C1 (7566)	S5 (5603)	PO3 (5297)
Two causals Variables	CS1+RE1 (7320)	C1+CS1 (7025)	C1+RE1 (6501)	CS1+S5 (5191)	CS1+PO3 (5148)
Three causals Variables	C1+CS1+RE1 (6137)	C1+CS1+PO3 (4924)	C1+CS1+S5 (4776)	CS1+RE1+S5 (4762)	C1+RE1+S5 (4653)
Four causals variables	C1+CS1+PO3+RE1 (4489)	C1+CS1+RE1+S5 (4451)	C1+CS1+PO3+S5 (3569)	CS1+PO3+RE1+S5 (3509)	C1+PO3+RE1+S5 (3442)

The causal variables considered in each of the scenarios analysed (1.1, 2.1, and 3.1) are characterised by variables that tend to have a low variability between different mines, workplaces, and accident groups, among other characteristics. This fact means that they have few classes (usually less than 6) and a high concentration of accidents in 1 or 2 classes, normally higher than 50 % of the instances. Thus, in scenario 1.1 the frequency of each causal variable varies between 94.24 % (R1) and 72.34 % (R5) of the instances in the 5 best rules, while it ranges between 84.86 % (R1) and 38.08 % (R5) in scenario 2.1 and between 89.08 % (R1) and 53.82 % (R5) in scenario 3.1.

The results obtained are very similar for the three scenarios, as the 5 most frequent classes of causal variables are the same (C1, CS1, PO3, RE1, and S5). Only the order of these variables varies according to the number of instances in which they appear. Thus, the most frequent causal variable in the three scenarios is the variable “Contractual Status" of class 1 (CS1: the injured worker belongs to the company that owns the mine “own worker”). On the other hand, the variables that appear less frequently are the size of the class 5 mine (S5: mines with 100 and 499 employees) for scenarios 1.1 and 2.1 and the variable relating to the type of class 3 Prevention Organisation (PO3: own prevention service) for scenario 3.1. These changes in the causal variables imply minimal changes in the association rules of 3 and, mainly, 4 variables of the 3 scenarios, being only changes in the ranking of the 5 best association rules of each case. This low variability of the classes of causal variables, as well as of the association rules extracted for the 3 scenarios, is consistent with what has been indicated above. In this way, the first accident rule of association of the 3 scenarios (1.1, 2.1, and 3.1) is the same. It is characterised by the fact that the injured worker was employed by the company owning the underground mining centre where the accident occurred, with a full-time permanent contract, with its own preventive organisation system, and with an occupational risk assessment carried out at the site where the accident occurred.

Based on the obtained results, the percentage of accidents with the variable contractual status of class 1 (CS1) is 94.24 %, 84.86 %, and 89.08 % for scenarios 1.1, 2.1, and 3.1, respectively. Consequently, the percentage of accidents with the variable indicated of class 0 (CS0) external workers is 5.76 %, 15.14 %, and 10.92 %, respectively. Likewise, the average percentage of own workers and external workers in Spanish underground mining has been calculated from the annual Spanish mining statistics within the period 2003–2021. Obtaining a value of 66 % and 34 % for own workers and for external workers, respectively. Thus, the average risk index for the period indicated can be calculated for the two groups of workers. The risk index is indicative of the incidence of accidents among different groups or subpopulations [52], and it is defined as the ratio of the percentage of injured workers, of a given subpopulation, to the percentage of the total workforce represented by this subpopulation, Equation (1). A risk index = 1 corresponds to an average incidence rate of work-related accidents, whereas a value greater than 1 indicates a higher incidence for the group and a value smaller than 1 means a lower incidence.

(1) $$\text{Risk}\;\text{Index}=\frac{\text{Accidents}\;\left(\%\right)}{\text{Workers}\;\left(\%\right)}$$

For scenarios 1.1, 2.1, and 3.1, the risk index for variable CS1 own workers is 1.43, 1.29, and 1.35 respectively, while for the external workers, it is 0.17, 0.45, and 0.32, respectively. These results indicate that the own workers in the Spanish underground mining had a significantly higher incidence of accidents than external workers in the period 2003–2021. This is not in line with other previous studies, which indicate that an increase in the use of subcontractors by the companies owning the mining centres usually means an increase in the number of accidents since it is easy for errors to occur in the coordination of business activities between the owner company and the subcontractors [53]. Further research should be done to analyse the specific characteristics of these results.

Concerning the variable risk evaluation in class 1 (RE1: The mining company has carried out a risk assessment at the site of the accident), an exact value for the percentage of mining companies that carry out risk assessments at all workplaces in their respective mining operations is not known. However, several factors suggest that the percentage must be very high. One is the entry into force of legislation in Spain requiring companies to carry out risk assessments in all workplaces. For the Spanish mining sector, “Royal Decree 1389/1997, of 5 September, approving the minimum provisions for the protection of the health and safety of workers in mining activities” and “Order ITC/101/2006, of 23 January, on the Health and Safety Document”; and its update with “Order TED/252/2020, of 6 March”. Another factor is the knowledge that the authors of this study have of the mining sector, based on their experience. Thus, it is estimated that 80 % of Spanish underground mines complied with the requirement to carry out a risk assessment of all workplaces in the period 2003–2021. From this, the risk index can be calculated taking into account that the percentage of accidents for each scenario of the variable RE1 is 85.40 %, 65.96 %, and 82.44 % for scenarios 1.1, 2.1, and 3.1, respectively. The risk index is 1.00, 0.776, and 1.03 for the accidents of the three scenarios. These results indicate that the accidents with RE1 in scenarios 1.1 and 3.1 had an average incidence rate, while there is a significantly lower incidence rate in scenario 2.1. However, it is important to reflect on these results. In principle, in a workplace where a risk assessment has been carried out and corrective or preventive measures have been taken, the risk of accidents should be reduced to a minimum. For this reason, it is surprising that in all the scenarios considered there is such a high percentage of accidents where it is stated that a risk assessment was carried out at the workplace where the accident occurred (RE1). This may indicate some kind of malfunction in the company's occupational risk prevention management system.

### 3.2 Scenarios 1.2, 2.2 and 3.2

**Fig. 5:**
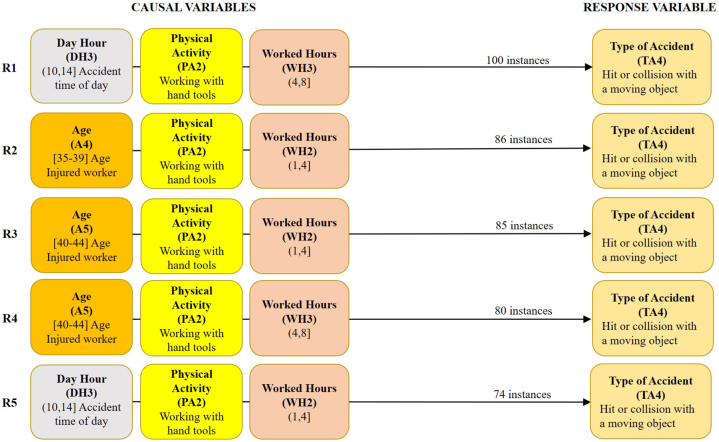
The 5 best association rules (R1 to R5) with four causal variables for scenario 1.2. Previous Cause (PC) = 3: Slip, fall, or collapse of a material object falling on the worker from an upper or a lateral position. Material agent (MA) = 3: Underground tunnels or drifts. Minimum support required to find all sets of frequent items = 10 %.

**Fig. 6:**
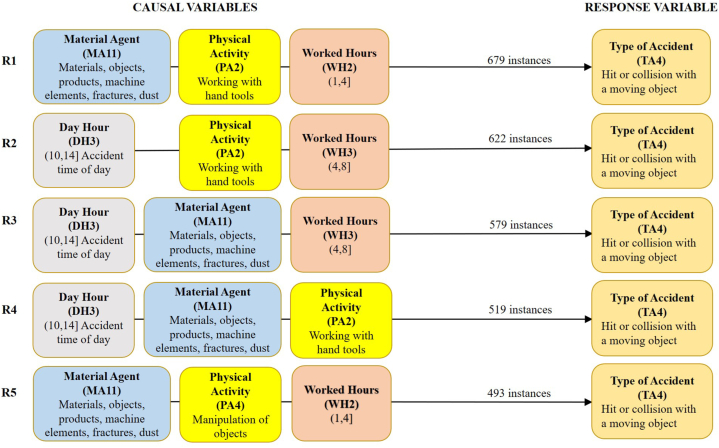
The 5 best association rules (R1 to R5) with three causal variables for scenario 2.2. Previous Cause (PC) = 3: Slip, fall, or collapse of a material object falling on the worker from an upper or a lateral position. Material Agent (MA)≠3: Underground tunnels or drifts. Minimum support required to find all sets of frequent items = 10 %.

**Fig. 7:**
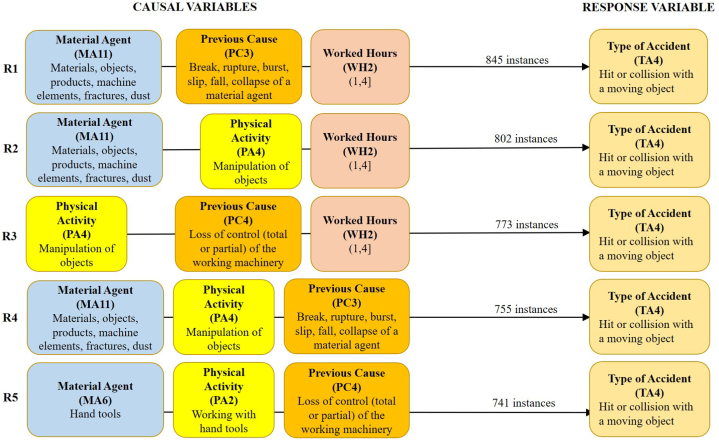
The 5 best association rules (R1 to R5) with three causal variables for scenario 3.2. Minimum support required to find all sets of frequent items = 8 %.

These scenarios include the accidents specific to each of their subgroups, with the causal or predictive variables Age (A), Day Hour (DH), Experience (E), Physical Activity (PA), Week Day (WD), and Work Hours (WH). Similarly, scenario 2.2 includes an additional causal variable, Material Agent (MA); and scenario 3.2 includes two additional causal variables, Material Agent (MA) and Deviation or Previous Cause (PC). Table 5, Table 6, Table 7 summarise the 5 best rules of association for one, two, or three causal variables, and Fig. 5, Fig. 6, Fig. 7 show the 5 best rules with the combination of the three variables.

**Table 5:** The best five first association rules for one, two, or three causal variables for scenario 1.2 with 781 accidents or instances.

	R1	R2	R3	R4	R5
One causal variable	PA2 (439)	WH2 (421)	WH3 (325)	DH3 (288)	A5 (280)
Two causals Variables	PA2+WH2 (224)	PA2 +WH3 (197)	DH3+PA2 (175)	A5+PA2 (172)	DH3+WH3 (165)
Three causals variables	DH3+PA2+WH3 (100)	A4+PA2+WH2 (86)	A5+PA2+WH2 (85)	A5+PA2+WH3 (80)	DH3+PA2+WH2 (74)

**Table 6:** The best five first association rules for one, two, or three causal variables for scenario 2.2 with 4906 accidents or instances.

	R1	R2	R3	R4	R5
One causal variable	MA11 (2656)	WH2 (2525)	PA2 (2465)	WH3 (2118)	DH3 (1855)
Two causals Variables	MA11+WH2 (1396)	MA11+PA2 (1298)	PA2+WH2 (1281)	DH3+WH3 (1127)	MA11+WH3 (1117)
Three causals variables	MA11+PA2+WH2 (679)	DH3+PA2+WH3 (622)	DH3+MA11+WH3 (579)	DH3+MA11+PA2 (519)	MA11+PA4+WH2 (493)

**Table 7:** The best five first association rules for one, two, and three causal variables for scenario 3.2 with 9841 accidents or instances.

	R1	R2	R3	R4	R5
One causal variable	WH2 (5026)	PC4 (4100)	WH3 (3942)	PA2 (3632)	PA4 (3534)
Two causals Variables	PC4+WH2 (2048)	PA4+WH2 (1840)	PA2+WH2 (1822)	PC3+WH2 (1800)	DH3+WH3 (1707)
Three causals variables	MA11+PC3+WH2 (845)	MA11+PA4+WH2 (802)	PA4+PC4+WH2 (773)	MA11+PA4+PC3 (755)	MA6+PA2+PC4 (741)

The 8 causal variables considered in each of the scenarios analysed (1.2, 2.2, and 3.2) are characterised by the fact that they tend to be highly variable since they define aspects that can vary greatly between workplaces, workers, previous causes just before the accident, etc. This means that they usually have more than 5 classes and with a moderate concentration of accidents in 2 or more classes, usually less than 50 % of the instances. Thus, in scenario 1.2, the frequency of each causal variable varies between 56.21 % (R1) and 35.85 % (R5) of the instances in the 5 best rules; in scenario 2.2, between 54.14 % (R1) and 37.81 % (R5); and in scenario 3.2, between 51.07 % (R1) and 35.91 % (R5).

The results obtained show a higher variability of the association rules compared to scenarios 1.1, 2.1, and 3.1. Likewise, the causal variables worked hours (WH) and physical activity (PA) appear in the top 5 causal variables in all scenarios (1.2, 2.2, and 3.2). In addition, the variable WH appears twice in all scenarios and PA appears twice in scenario 3.2. Consequently, one or two of these indicated causal variables (WH and PA) are present in all 2- and 3-variable association rules.

Regarding the association rules with 3 causal variables, the variable WH is present in class 2, WH2: (1 h, 4 h], in the best association rules with 3 causal variables R2, R3 and R5 of scenario 1.2; in R1 and R5 of scenario 2.2; and in R1, R2 and R3 of scenario 3.2. WH is also present in class 3, WH3: (4 h, 8 h], in R1 and R4 of scenario 1.2; and in R2 and R3 of scenario 2.2.

In summary, more than 50 % of the accidents in the three scenarios analysed occurred between the first 1 and 4 h of work. It should be noted that during this period the miners often take a break to eat and drink. It is known that after a meal, the level of alertness required to perform their respective tasks properly when they return to work may be reduced [54]. In this respect, those responsible for the management of occupational risk prevention should warn against this during training and information sessions. Concerning the variable PA, it appears in class 4 (PA4: Manipulation of objects) and mainly in class 2 (PA2: Working with hand tools). PA2 is present in the 5 best rules with 3 causal variables of scenario 1.2, in 3 rules of scenario 2.2, and in rule R5 of scenario 3.2, where the variable PA4 is present in R2, R3, and R4. In short, in more than 50 % of the accidents in scenarios 1.2 and 2.2, the injured workers were working with hand tools, which is in line with other studies [28,55].

It is important to note that the variable previous cause of class 3 (PC3: slip, fall, or collapse of a material object falling on the worker from above or from the side) is present in all the accidents in scenarios 1.2 and 2.2, as is the variable material agent, also of class 3 (MA3: underground tunnels or drifts) for scenario 1.2. However, it was decided not to include them in the tables and figures of the results for each scenario, as they were not taken into account in the Weka calculation process, since there is only one class for each variable. This allowed other causal variables to appear in the association rules, allowing for more detail on the circumstances leading up to the accident.

As far as scenario 1.2 is concerned, which includes the 781 accidents in which the anomalous event that caused the accident was the slip, fall, or collapse of a material object falling on the worker from an upper position or a lateral position from a mine drift, it should be noted that, apart from the variables WH and PA, variables of age of the injured worker in class 4, A4: [35,39], and mainly in class 5, A5: [40,44], appear in the association rules. Also, the variable of the time of day when the accident occurred in class 3, DH3: [10,14]. Thus, the first association rule with three causal variables (R1), in scenario 1.2, was given by the detachment of a rock or part of the roof or side wall of a mine drift, in the time slot of the day from 10:00 a.m. to 14:00 p.m., on a worker who was working with a hand tools in the area of influence of the detached objects, between 4 and 8 h of his/her working shift. The second association rule (R2) is the same as the previous one except that the time of day of the accident is not known and the injured worker is between 35 and 39 years old, and having worked between 1 and 4 h of his/her working shift. The third rule (R3) is the same as the second, but the age of the worker is between 40 and 44 years. In this scenario.

Scenario 2.2 includes the 4906 accidents in which the anomalous event causing the accident was a slip, fall, or collapse of a material object falling on the worker, as in the accidents in scenario 1.2. The fundamental difference here is that falling rocks or parts of the roof and walls of mine drifts are not covered. In short, accidents involving falling objects and materials falling on a worker that are not related to geotechnical collapses are included. The results of Table 6 and Fig. 6 show that the material agent of class 11 (MA11: Materials, objects, products, machine elements, fractures, dust) is the material agent that appears in 46.27 % of the instances of the association rules of the three causal variables R1, R3, R4, and R5.

In addition to the causal variables already mentioned (MA11, WH2, WH3, PA2, and PA4), the variable Day Time of class 3, DH3: (10,14), also stands out, appearing in 37.81 % of the instances, and in 3 of the first 5 association rules of three causal variables, concretely, in rules R2, R3, and R4. Thus, the first association rule with three causal variables (R1) in scenario 2.2 was given by the fall of an element (material, object, product, machine part, etc.) from an upper or lateral position on the worker, who was working with hand tools in the area of influence of the fallen element, between 1 and 4 h of his/her working shift. The second association rule (R2) is the same as the previous one, except that the agent is not known, the accident occurred in the 10:00 to 14:00 time slot of the day, and the worker had been working for (4–8] hours. The third rule (R3) is the same as the first, but the physical activity of the worker is not known and the accident occurred in the 10:00 to 14:00 time slot of the day.

Scenario 3.2 includes the remaining 9841 type 4 accidents out of a total of 15,528 in Spanish underground mining that occurred during the period 2003–2021. In this scenario, no conditions are set regarding the causal variables, so that in the calculation process using Weka, 8 causal variables were considered, instead of 6 in scenario 1.2 and 7 in scenario 2.2. Therefore, PC3 (Break, rupture or burst, slip, fall, collapse of a material agent) and PC4 (Total or partial loss of control of work equipment) appear as the association rules. PC4 is the second class variable most frequent, occurring in 41.66 % of the 9841 accidents. On the other hand, PC3 does not appear at all, whereas it does in the rules with 2 and 3 causal variables. The material agent does not appear as one of the 5 most frequent variables, but it does appear in 4 of the 3 variable association rules, in R1, R2, and R3 in class 11, and in R5 in class 6 (M6: hand tools). Thus, the first association rule with three causal variables (R1) of scenario 3.2 was given by the break, rupture, or burst of a material agent of an element (material, object, product, machine part, etc.) affecting the worker who was working in the zone of influence of the breaking, rupturing or bursting element between 1 and 4 h from the start of the work shift. The second association rule (R2) is the same as the previous one, except that in this rule the anomalous fact or previous cause is not known, but the physical activity of the worker is known, which is the manipulation of objects. The third rule (R3) is characterised by the worker hitting or colliding with a machine due to loss of control (total or partial) over which the worker has had control for 1–4 h from the start of the working shift.

Results from three scenarios suggest that increased mechanisation and automation of tasks could lead to a significant reduction in the number of accidents, mainly in the subgroup of accidents 1 and 2 (scenarios 1.1, 1.2, 2.1, and 2.2). Technical and occupational risk prevention managers should, therefore, implement mechanisation and automation processes as soon as possible whenever it is feasible. In this way, the use of wireless sensor network (WSN) technology has the potential to improve the health and safety monitoring of miners and operators, and could be a solution given that the underground environment of mines is very complex and hazardous [56]. Besides, continuous monitoring of roof and wall movements in underground spaces could also reduce the accident and incident levels.

In the case of subgroup 1 (scenarios 1.1 and 1.2) in which, for geological reasons, rocks or parts of the roof and walls of the mine drift fall over a worker, it is crucial to have well-established procedures to carry out daily supervision of the areas of the mine drift where this type of risk may exist. It is important to note that this supervision is carried out by a process as automated as possible to reduce the exposure of workers, such as autonomous scanners, drones, etc. [[57], [58], [59]], determining when is safe to access or which areas must be closed or further actions are required, such as the usage of heavy protected equipment or the usage of additional support techniques.

Finally, it should be noted that the accidents in subgroup 1 (scenarios 1.1 and 1.2) are directly attributable to poor environmental conditions, in particular geological causes, which, as it has been seen, had more serious consequences than the accidents in the other subgroups. Only in cases where the injured worker failed to comply with a very clear rule, such as a prohibition on working in a particular part of the mine because of rockmass bad conditions, could the accident be attributed to human error. In subgroups 2 and 3, although accidents are influenced by the specific mine environment, the results obtained suggest that a higher percentage of accidents would be attributable to human error.

### 3.3 Limitations

Data processing using the Apriori algorithm, with the Weka software, is a good tool for discovering behavioural patterns or association rules in data mining of occupational accidents. The importance of selecting and classifying causal variables that indicate different characteristics of the injured worker or the workplace, among others, has been demonstrated. In the present study, it was considered necessary to divide the 13 selected causal variables into 2 groups, a group of 5 variables and a second group of 8 variables. In the first group, the accidents or instances are essentially concentrated in 1 or 2 classes and define aspects that are largely consistent with the general characteristics of Spanish underground mining. Moreover, a large number of these aspects are directly or indirectly influenced by legislative or regulatory causes, as indicated at the end of section 2.2. Therefore, if this study had been carried out with all 13 variables in the same treatment, the results would have been practically the same as those obtained with the 5 variables of the first group, and all the information provided by the treatment with the second group of 8 causal variables considered would have been unknown. Therefore, in order to obtain a much better and deeper understanding of the data, it is essential to be able to detect the existence of the variables in the first group and to, either discard them from the processing or carry out another processing with them if there is a minimum number of them.

Another limitation (in this case due to the accident database) is the inability to determine whether an accident type (TA) with an antecedent cause (PC) is due to deficiencies in the preventive organisation of the mine where the accident occurred, or to human error of the workers. In addition, results from section 3.1 have shown that a very high percentage of accidents occurred in workplaces where a risk assessment had been carried out, which could mean that the company's preventive organisation system was not working properly. This cannot be established at present because the current accident database only allows to know whether the workplace where the accident occurred had been subject to a risk assessment or not. It does not make it possible to identify any type of malfunction in the company's occupational risk prevention system, such as inadequate or outdated risk assessments, failure to take the necessary preventive or corrective measures to eliminate the identified risks, etc. Therefore, the Spanish Public Administration Accident Database should include, in addition to the risk assessment variable (RE), other fields that could more reliably reflect the quality of an occupational risk prevention management system, as well as possible human errors.

## 4 Conclusions

The behavioural patterns or association rules of the 15,548 type 4 accidents that occurred in Spanish underground mines during the period 2003–2021 have been identified. In order to extract as much information as possible on the genesis of these accidents, they have been divided into 3 subgroups and processed in 2 different ways, depending on the input or causal variables used in each case. This allowed the study to be carried out for 6 different scenarios: 1.1, 2.1, and 3.1 with 5 causal variables used in the processing; and, on the other hand, 1.2, 2.2 and 3.2 with 6, 7, and 8 causal variables used, respectively.

For scenarios 1.1, 2.1, and 3.1, the results have shown that the first accident rule of association of the 3 scenarios is the same. It is characterised by the fact that the injured worker was employed by the company owning the underground mine where the accident occurred, with a full-time permanent contract, its own preventive organisation system and an occupational risk assessment carried out at the site where the accident occurred. The results also show that the group of workers directly employed by the companies owning the mine had a much higher incidence of accidents at work than the group of subcontracted or external workers. Further research should be done to determine this incidence ratio difference.

Regarding scenarios 1.2, 2.2, and 3.2 with 6, 7, and 8 causal variables, respectively, the association rules have changed much more. This is because the 8 variables used are characterised by greater variability between mines, workplaces, workers and different times of the day and week. Thus, the first rule with 3 causal variables obtained for scenario 1.2 is given by the detachment of a rock or part of the roof or side wall of a mine drift in the time slot of the day from 10:00 a.m. to 14:00 p.m., affecting a worker using hand tools in the area of influence of the detached objects, between 4 and 8 h of his/her working shift. For scenario 2.2, the first rule with three causal variables obtained is given by the fall of an element (material, object, product, machine part, etc.) from an upper or lateral position on the worker, who was using hand tools in the area of influence of the fallen element, between 1 and 4 h of his/her working shift. While for scenario 3.2, the first rule is given by the break, rupture or burst of a material agent (material, object, product, machine part, etc.) affecting the worker who was at the area of influence of the breaking, rupturing or bursting between 1 and 4 h from the start of the work shift.

The results also showed the importance of mechanisation and automation of all possible tasks, as it could be seen that the worker was performing a task with hand tools, mainly in scenarios 1.2 and 2.2, which could lead to a very significant reduction in occupational accidents.

Finally, scenario 3.2 includes accidents due to breakage, rupture and bursting of the material agent, and accidents due to loss of control of a hand tool with which the injured worker was working. This could be due to failures in the preventive organisation of the company (inadequate training of workers, inadequate maintenance of work equipment, etc.), although human error cannot be ruled out. In this sense, it would be essential to extend the fields of the accident database in order to differentiate between them.

## CRediT authorship contribution statement

**Lluís Sanmiquel:** Writing – original draft, Validation, Supervision, Investigation, Data curation, Conceptualization. **Josep M. Rossell:** Writing – original draft, Methodology, Investigation, Formal analysis. **Marc Bascompta:** Writing – review & editing, Data curation, Conceptualization. **Carla Vintró:** Visualization, Investigation. **Mohammad Yousefian:** Writing – review & editing, Visualization.

## Declaration of competing interest

The authors declare that they have no known competing financial interests or personal relationships that could have appeared to influence the work reported in this paper.
